# Genetic Variants Associated With Neurodegenerative Diseases Regulate Gene Expression in Immune Cell CD14+ Monocytes

**DOI:** 10.3389/fgene.2018.00666

**Published:** 2018-12-18

**Authors:** Jing-yi Sun, Ya-jun Hou, Yan Zhang, Longcai Wang, Lidong Liu, Bao-liang Sun, Hui Yuan

**Affiliations:** ^1^Wonju Severance Christian Hospital, Yonsei University Wonju College of Medicine, Wonju, South Korea; ^2^Key Laboratory of Cerebral Microcirculation, Department of Neurology, Affiliated Hospital of Taishan Medical University, Universities of Shandong, Taian, China; ^3^Department of Pathology, The Affiliated Hospital of Weifang Medical University, Weifang, China; ^4^Department of Anesthesiology, The Affiliated Hospital of Weifang Medical University, Weifang, China; ^5^Brain Research Centre, University of British Columbia, Vancouver, BC, Canada

**Keywords:** genome-wide association study, neurodegenerative disease, eQTLs, CD14+ monocytes, Alzheimer's disease

## Abstract

Until now, large-scale genome-wide association studies have identified 94 genes associated with Alzheimer's disease, Parkinson's disease, and multiple sclerosis. Expression quantitative trait locus (eQTL) analysis showed that six genetic variants around six of these 94 genes could drive both disease susceptibility and altered expression of six nearby genes including CD33 (rs3865444), PILRB (rs1476679), NUP160 (rs10838725), LRRK2 (rs76904798), RGS1 (rs1323292), and METTL21B (rs701006). However, two of these six genetic variants rs1476679 and rs76904798 variants could regulate the expression of PILRB and LRRK2 only in the human monocyte-derived microglia-like (MDMi) cells, but not in human peripheral blood monocytes. Here, we aim to verify these findings using another two eQTL datasets in human peripheral blood immune cell CD14+ monocytes. The results that showed that rs1476679 and rs76904798 variants or their proxy variants could significantly regulate the expression of PILRB and LRRK2 in immune cell CD14+ monocytes and human peripheral blood. We believe that these findings provide important supplementary information about the regulatory mechanisms by which both variants influence PILRB and LRRK2 gene expression and neurodegenerative disease risk.

## Introduction

It is known that Alzheimer's disease, Parkinson's disease and multiple sclerosis are three common neurodegenerative diseases (Liu et al., 2012, 2016, 2017b,c,d,e, 2018c; Beecham et al., 2013; Chang et al., 2017; Jun et al., 2017). In recent years, large-scale genome-wide association studies (GWAS) have identified some neurodegenerative disease risk variants (Liu et al., 2012, 2016, 2017b,c,d,e, 2018b; Beecham et al., 2013; Zhang et al., 2015, 2018; Li et al., 2016; Chang et al., 2017; Jiang et al., 2017; Jun et al., 2017). In 2013, the International Genomics of Alzheimer's Project (IGAP) conducted a meta-analysis of Alzheimer's disease GWAS datasets including 17,008 Alzheimer's disease cases and 37,154 controls in stage 1, and 8,572 Alzheimer's disease cases and 11,312 controls in stage 2. The meta-analysis of 74,046 individuals in stage 1 and stage 2 identified 11 new susceptibility loci for Alzheimer's disease (Lambert et al., 2013). In 2014, Nalls et al. conducted a large-scale meta-analysis of Parkinson's disease GWAS data including up to 13,708 PD cases and 95,282 control from 15 independent GWAS datasets of European descent, and identified six new Parkinson's disease risk loci (Nalls et al., 2014). In 2017, Chang et al. conducted a meta-analysis of Parkinson's disease GWAS data including 26,035 cases and 403,190 controls, and identified 17 novel Parkinson's disease risk loci (Chang et al., 2017).

Evidence shows that genetic variants could modify gene expression and cause disease risk (Liu et al., 2017d, 2018a,b,d). In a recent study, Ryan and colleagues developed a human monocyte-derived microglia-like (MDMi) cellular model to assess the effects of neurodegenerative disease variants (Ryan et al., 2017). Using this model system, Ryan and colleagues conducted an expression quantitative trait locus (eQTL) analysis to examine 94 genes associated with Alzheimer's disease, Parkinson's disease, and multiple sclerosis (Ryan et al., 2017). One eQTL analysis was performed using MDMi cells from 95 young, healthy subjects of European ancestry (Ryan et al., 2017). The other eQTL analysis was performed using human peripheral blood monocytes derived from 211 young, healthy subjects of European ancestry (Ryan et al., 2017). They identified that genetic variants could drive both disease susceptibility and altered expression of six nearby genes including CD33 (rs3865444), PILRB (rs1476679), NUP160 (rs10838725), LRRK2 (rs76904798), RGS1 (rs1323292), and METTL21B (rs701006) (Ryan et al., 2017). However, rs1476679 and rs76904798 variants could regulate the expression of PILRB and LRRK2 only in the MDMi cells, but not in human peripheral blood monocytes (Ryan et al., 2017). Here, we aim to further verify their findings using multiple eQTL datasets in human peripheral blood immune cell CD14+ monocytes.

## Materials and Methods

### Functional Analysis

It is described that enhancers are DNA regulatory sequences, and could regulate tissue-specific gene expression (Ong and Corces, 2011; Shlyueva et al., 2014). Hence, we first performed an enhancer analysis using HaploReg (version 4.1) to evaluate the overlap of these six variants with predicted enhancers in each reference epigenome data from Roadmap Epigenomics project (Ward and Kellis, 2016). HaploReg (version 4.1) defined the enhancers using four different methods including the 15-state core model, the 25-state model incorporating imputed epigenomes, the H3K4me1/H3K4me3 peaks and the H3K27ac/H3K9ac peaks (Ward and Kellis, 2016). In brief, both the core 15-state model and the 25-state model are based on the imputed marks, H3K4me1/H3K4me3 is based on peaks from H3K4me1 and H3K4me3, and H3K27ac/H3K9ac is based on peaks from H3K27ac and H3K9ac (Ward and Kellis, 2016). In addition to enhancer analysis, we also conducted a promoter analysis and DNAse analysis. More detailed information has been described in recent study (Liu et al., 2018b).

### eQTL Analysis

The first eQTL dataset in monocytes is from the International Human Epigenome Consortium (IHEC) (Chen et al., 2016). The IHEC consortium performed an eQTL analysis in three immune cell types (CD14+ monocytes, CD16+ neutrophils, and naive CD4+ T cells) from up to 197 individuals (Chen et al., 2016). Data from this project was produced in different institutes. Peripheral blood mononuclear cells were isolated from donors at University of Cambridge (Chen et al., 2016). The second eQTL dataset in monocytes (CD14+ monocytes) is from a recent study conducted by Fairfax et al. (2014). In the original study, primary monocytes from 432 healthy Europeans were exposed to interferon-γ (IFN-γ) or differing durations of lipopolysaccharide (LPS) (Fairfax et al., 2014). Then the impact of immune stimulation upon regulatory variant activity was systematically evaluated by an eQTL analysis (Fairfax et al., 2014). In brief, this eQTL analysis tested 609,704 genetic variants and expression data for 15,421 probes for 414 individuals in the naive state, 367 individuals after exposure to IFN-γ, 322 individuals after 24-h LPS, and 261 individuals after 2-h LPS (Fairfax et al., 2014).

In both eQTL datasets, genetic variants mapping to within 1 Mb (on each side) of each tested gene, were tested their association with gene expression using a linear regression analysis (Fairfax et al., 2014; Chen et al., 2016). Here, we utilize the summary results from the corresponding studies to evaluate the potential association of six genetic variants with the expression of their nearby genes. If any variant is not available in above datasets, we used HaploReg (version 4) to identify it proxy variants based on the linkage disequilibrium (LD) information from the 1000 Genomes Project (EUR) (Ward and Kellis, 2012). We defined the genetic variants tagged by any genetic variant with *r*^2^> = 0.8 (Ward and Kellis, 2012). In addition to the monocytes, we also evaluated the association of six genetic variants with gene expression using two eQTL datasets in human peripheral blood including 2,116 and 5,257 samples, respectively (Joehanes et al., 2017; Zhernakova et al., 2017).

## Results

### Enhancer Analysis

The enhancer analysis using the 15-state core model, the 25-state model, the H3K4me1/H3K4me3 model and the H3K27ac/H3K9ac model showed that these 6 genetic variants are predicted to be mainly located in enhancers of blood cell types. These findings indicate that these 6 genetic variants are likely to regulate gene expression in the blood cell types, especially CD14+ monocytes. In brief, rs1476679 variant is predicted to be located in enhancers of monocytes-CD14+ RO01746 Primary Cells using the H3K4me1/H3K4me3 model and the H3K27ac/H3K9ac model (Table 1). The rs76904798 variant is predicted to be located in enhancers of monocytes-CD14+ RO01746 Primary Cells using the 15-state core model, the H3K4me1/H3K4me3 model and the H3K27ac/H3K9ac model. To validate the enhancer analysis, we then performed an eQTLs analysis using multiple datasets in CD14+ monocytes.

**Table 1:** Enhancer analysis of six neurodegenerative disease variants using HaploReg (version 4.1).

**SNP**	**15-state core model**	**25-state model**	**H3K4me1/H3K4me3 model**	**H3K27ac/H3K9ac model**
rs1323292	Primary T cells from cord blood, Primary T helper memory cells from peripheral blood 2, Primary T helper naive cells from peripheral blood, Primary T helper naive cells from peripheral blood, Primary T helper memory cells from peripheral blood 1, Primary T helper cells PMA-I stimulated, Primary T helper cells from peripheral blood, Primary T regulatory cells from peripheral blood, Primary T CD8+ naive cells from peripheral blood, Primary T CD8+ memory cells from peripheral blood	Primary T cells from cord blood, Primary T cells from peripheral blood, Primary hematopoietic stem cells short term culture, Primary T helper memory cells from peripheral blood 2, Primary T helper naive cells from peripheral blood, Primary T helper naive cells from peripheral blood, Primary T helper memory cells from peripheral blood 1, Primary T helper cells PMA-I stimulated, Primary T helper 17 cells PMA-I stimulated, Primary T helper cells from peripheral blood, Primary T regulatory cells from peripheral blood, Primary T cells effector/memory enriched from peripheral blood, Primary Natural Killer cells from peripheral blood, Primary T CD8+ naive cells from peripheral blood, Primary T CD8+ memory cells from peripheral blood, Primary hematopoietic stem cells G-CSF-mobilized Female, Primary mononuclear cells from peripheral blood, Fetal Thymus, Dnd41 TCell Leukemia Cell Line, GM12878 Lymphoblastoid Cells, K562 Leukemia Cells	Adipose Derived Mesenchymal Stem Cell Cultured Cells, Primary T cells from cord blood, Primary T cells from peripheral blood, Primary T helper memory cells from peripheral blood 2, Primary T helper naive cells from peripheral blood, Primary T helper naive cells from peripheral blood, Primary T helper memory cells from peripheral blood 1, Primary T helper cells PMA-I stimulated, Primary T helper 17 cells PMA-I stimulated, Primary T helper cells from peripheral blood, Primary T regulatory cells from peripheral blood, Primary T cells effector/memory enriched from peripheral blood, Primary T CD8+ naive cells from peripheral blood, Primary T CD8+ memory cells from peripheral blood, Cortex derived primary cultured neurospheres, Foreskin Fibroblast Primary Cells skin02, Colon Smooth Muscle, Duodenum Mucosa, Duodenum Smooth Muscle, Rectal Mucosa Donor 29, Rectal Mucosa Donor 31, Small Intestine, Thymus, Dnd41 TCell Leukemia Cell Line, NHLF Lung Fibroblast Primary Cells	Primary T cells from peripheral blood, Primary T helper memory cells from peripheral blood 2, Primary T helper naive cells from peripheral blood, Primary T helper naive cells from peripheral blood, Primary T helper memory cells from peripheral blood 1, Primary T helper cells PMA-I stimulated, Primary T helper 17 cells PMA-I stimulated, Primary T helper cells from peripheral blood, Primary T regulatory cells from peripheral blood, Primary T cells effector/memory enriched from peripheral blood, Primary T CD8+ memory cells from peripheral blood, Primary mononuclear cells from peripheral blood, Colon Smooth Muscle, Duodenum Smooth Muscle, Esophagus, Fetal Thymus, Rectal Smooth Muscle, Stomach Smooth Muscle, Thymus, Dnd41 TCell Leukemia Cell Line.
rs701006	Cortex derived primary cultured neurospheres, Foreskin Melanocyte Primary Cells skin03, Brain Cingulate Gyrus, Brain Hippocampus Middle, Brain Inferior Temporal Lobe	Cortex derived primary cultured neurospheres, Ganglion Eminence derived primary cultured neurospheres, Brain Angular Gyrus, Brain Anterior Caudate, Brain Cingulate Gyrus, Brain Hippocampus Middle, Brain Inferior Temporal Lobe, Brain_Dorsolateral_Prefrontal_Cortex, Brain Substantia Nigra	hESC Derived CD56+ Ectoderm Cultured Cells, Primary T helper memory cells from peripheral blood 2, Cortex derived primary cultured neurospheres, Foreskin Melanocyte Primary Cells skin03, Brain Angular Gyrus, Brain Anterior Caudate, Brain Cingulate Gyrus, Brain Hippocampus Middle, Brain Inferior Temporal Lobe, Brain Substantia Nigra, Placenta, Lung, Small Intestine	Primary T helper memory cells from peripheral blood 2, Primary T helper naive cells from peripheral blood, Foreskin Fibroblast Primary Cells skin02, Foreskin Melanocyte Primary Cells skin03, Brain Angular Gyrus, Brain Anterior Caudate, Brain Cingulate Gyrus, Brain Hippocampus Middle, Brain Inferior Temporal Lobe, Brain_Dorsolateral_Prefrontal_Cortex, Brain Substantia Nigra, Right Ventricle, **Monocytes-CD14+ RO01746 Primary Cells**
rs1476679	hESC Derived CD184+ Endoderm Cultured Cells, Mesenchymal Stem Cell Derived Adipocyte Cultured Cells, Adipose Derived Mesenchymal Stem Cell Cultured Cells, Bone Marrow Derived Cultured Mesenchymal Stem Cells, Breast variant Human Mammary Epithelial Cells (vHMEC), Foreskin Fibroblast Primary Cells skin01, Foreskin Keratinocyte Primary Cells skin03, Fetal Thymus, Stomach Mucosa, GM12878 Lymphoblastoid Cells, HMEC Mammary Epithelial Primary Cells, HSMM Skeletal Muscle Myoblasts Cells, HSMM cell derived Skeletal Muscle Myotubes Cells, K562 Leukemia Cells, NH-A Astrocytes Primary Cells, Osteoblast Primary Cells	Mesenchymal Stem Cell Derived Adipocyte Cultured Cells, Adipose Derived Mesenchymal Stem Cell Cultured Cells, Breast variant Human Mammary Epithelial Cells (vHMEC), Mesenchymal Stem Cell Derived Chondrocyte Cultured Cells, Foreskin Fibroblast Primary Cells skin02, Foreskin Keratinocyte Primary Cells skin02, HSMM cell derived Skeletal Muscle Myotubes Cells, NHDF-Ad Adult Dermal Fibroblast Primary Cells, NHLF Lung Fibroblast Primary Cells	ES-I3 Cells, H1 Derived Mesenchymal Stem Cells, H9 Derived Neuronal Progenitor Cultured Cells, hESC Derived CD184+ Endoderm Cultured Cells, hESC Derived CD56+ Ectoderm Cultured Cells, hESC Derived CD56+ Mesoderm Cultured Cells, IMR90 fetal lung fibroblasts Cell Line, Mesenchymal Stem Cell Derived Adipocyte Cultured Cells, Adipose Derived Mesenchymal Stem Cell Cultured Cells, Bone Marrow Derived Cultured Mesenchymal Stem Cells, Breast variant Human Mammary Epithelial Cells (vHMEC), Primary monocytes from peripheral blood, Primary T cells from peripheral blood, Mesenchymal Stem Cell Derived Chondrocyte Cultured Cells, Foreskin Fibroblast Primary Cells skin01, Foreskin Fibroblast Primary Cells skin02, Foreskin Keratinocyte Primary Cells skin02, Foreskin Keratinocyte Primary Cells skin03, Brain Angular Gyrus, Colonic Mucosa, Fetal Thymus, Pancreas, Stomach Mucosa, GM12878 Lymphoblastoid Cells, HeLa-S3 Cervical Carcinoma Cell Line, HMEC Mammary Epithelial Primary Cells, HSMM Skeletal Muscle Myoblasts Cells, HSMM cell derived Skeletal Muscle Myotubes Cells, **Monocytes-CD14+ RO01746 Primary Cells**, NH-A Astrocytes Primary Cells, NHEK-Epidermal Keratinocyte Primary Cells, Osteoblast Primary Cells	hESC Derived CD56+ Mesoderm Cultured Cells, **Monocytes-CD14+ RO01746 Primary Cells**
rs10838725			H1 Derived Mesenchymal Stem Cells, hESC Derived CD56+ Mesoderm Cultured Cells, IMR90 fetal lung fibroblasts Cell Line, Mesenchymal Stem Cell Derived Adipocyte Cultured Cells, ES-UCSF4 Cells, Adipose Derived Mesenchymal Stem Cell Cultured Cells, Bone Marrow Derived Cultured Mesenchymal Stem Cells, Breast Myoepithelial Primary Cells, Breast variant Human Mammary Epithelial Cells (vHMEC), Primary monocytes from peripheral blood, Primary B cells from peripheral blood, Primary T cells from peripheral blood, Primary T helper memory cells from peripheral blood 2, Primary T helper naive cells from peripheral blood, Primary T helper naive cells from peripheral blood, Primary T helper memory cells from peripheral blood 1, Primary T helper cells PMA-I stimulated, Primary T helper 17 cells PMA-I stimulated, Primary T helper cells from peripheral blood, Primary T regulatory cells from peripheral blood, Primary T cells effector/memory enriched from peripheral blood, Primary Natural Killer cells from peripheral blood, Primary T CD8+ naive cells from peripheral blood, Primary T CD8+ memory cells from peripheral blood, Primary hematopoietic stem cells G-CSF-mobilized Female, Primary hematopoietic stem cells G-CSF-mobilized Male, Cortex derived primary cultured neurospheres, Ganglion Eminence derived primary cultured neurospheres, Foreskin Fibroblast Primary Cells skin01, Foreskin Fibroblast Primary Cells skin02, Foreskin Keratinocyte Primary Cells skin02,	H1 Derived Mesenchymal Stem Cells, hESC Derived CD56+ Ectoderm Cultured Cells, hESC Derived CD56+ Mesoderm Cultured Cells, Primary T helper memory cells from peripheral blood 2, Primary T helper naive cells from peripheral blood, Primary T helper cells PMA-I stimulated, Primary T helper 17 cells PMA-I stimulated, Primary T regulatory cells from peripheral blood, Primary hematopoietic stem cells G-CSF-mobilized Female, Foreskin Fibroblast Primary Cells skin01, Foreskin Keratinocyte Primary Cells skin03, Foreskin Melanocyte Primary Cells skin03, Adipose Nuclei, Brain Angular Gyrus, Brain Anterior Caudate, Brain Inferior Temporal Lobe, Brain_Dorsolateral_Prefrontal_Cortex, Colon Smooth Muscle, Pancreatic Islets, Psoas Muscle,
			Foreskin Keratinocyte Primary Cells skin03, Foreskin Melanocyte Primary Cells skin01, Foreskin Melanocyte Primary Cells skin03, Adipose Nuclei, Aorta, Liver, Brain Angular Gyrus, Brain Anterior Caudate, Brain Cingulate Gyrus, Brain Germinal Matrix, Brain Hippocampus Middle, Brain_Dorsolateral_Prefrontal_Cortex, Brain Substantia Nigra, Colonic Mucosa, Colon Smooth Muscle, Duodenum Mucosa, Duodenum Smooth Muscle, Fetal Adrenal Gland, Fetal Brain Male, Fetal Brain Female, Fetal Heart, Fetal Intestine Small, Pancreatic Islets, Fetal Lung, Fetal Muscle Trunk, Fetal Muscle Leg, Fetal Stomach, Fetal Thymus, Left Ventricle, Lung, Ovary, Pancreas, Psoas Muscle, Rectal Mucosa Donor 31, Rectal Smooth Muscle, Right Atrium, Right Ventricle, Sigmoid Colon, Skeletal Muscle Male, Skeletal Muscle Female, Small Intestine, Stomach Mucosa, Stomach Smooth Muscle, Spleen, A549 EtOH 0.02pct Lung Carcinoma Cell Line, HeLa-S3 Cervical Carcinoma Cell Line, HSMM cell derived Skeletal Muscle Myotubes Cells, **Monocytes-CD14+ RO01746 Primary Cells**, NH-A Astrocytes Primary Cells, NHEK-Epidermal Keratinocyte Primary Cells, NHLF Lung Fibroblast Primary Cells, Osteoblast Primary Cells	**Monocytes-CD14+ RO01746 Primary Cells**, NH-A Astrocytes Primary Cells, Osteoblast Primary Cells
rs76904798	Primary monocytes from peripheral blood, Primary neutrophils from peripheral blood, **Monocytes-CD14+ RO01746 Primary Cells**		Primary monocytes from peripheral blood, Primary neutrophils from peripheral blood, Primary B cells from cord blood, **Monocytes-CD14+ RO01746 Primary Cells**	**Monocytes-CD14+ RO01746 Primary Cells**
rs3865444	Primary monocytes from peripheral blood, Primary neutrophils from peripheral blood, Primary hematopoietic stem cells short term culture, Primary T regulatory cells from peripheral blood, Primary hematopoietic stem cells G-CSF-mobilized Female, Primary hematopoietic stem cells G-CSF-mobilized Male	Primary B cells from cord blood, Primary B cells from peripheral blood, Primary T cells from cord blood, Primary hematopoietic stem cells, Primary T regulatory cells from peripheral blood, Primary Natural Killer cells from peripheral blood, Primary hematopoietic stem cells G-CSF-mobilized Female, Primary mononuclear cells from peripheral blood, Duodenum Mucosa, Duodenum Smooth Muscle, Fetal Thymus, Dnd41 TCell Leukemia Cell Line, K562 Leukemia Cells, **Monocytes-CD14+ RO01746 Primary Cells**	Primary monocytes from peripheral blood, Primary neutrophils from peripheral blood, Primary hematopoietic stem cells, Primary hematopoietic stem cells short term culture, Primary T regulatory cells from peripheral blood, Primary Natural Killer cells from peripheral blood, Primary hematopoietic stem cells G-CSF-mobilized Female, Primary hematopoietic stem cells G-CSF-mobilized Male, Adipose Nuclei, Liver, Fetal Brain Male, Fetal Thymus, Rectal Mucosa Donor 29, Spleen, K562 Leukemia Cells, **Monocytes-CD14+ RO01746 Primary Cells**	iPS DF 6.9 Cells, Primary monocytes from peripheral blood, Primary T helper memory cells from peripheral blood 2, Primary T CD8+ memory cells from peripheral blood, Primary hematopoietic stem cells G-CSF-mobilized Female, Brain Anterior Caudate, Brain Hippocampus Middle, Brain Substantia Nigra, Lung, Spleen, K562 Leukemia Cells, **Monocytes-CD14+ RO01746 Primary Cells**

*CD14+ cells are bolded*.

### Promoter Analysis

All these four models including 15-state core model, the 25-state model, the H3K4me1/H3K4me3 model and the H3K27ac/H3K9ac model showed that the rs3865444 variant was predicted to be mainly located in promoter of blood cell types, especially in immune cell types. Three of these four models including the 15-state core model, the H3K4me1/H3K4me3 model and the H3K27ac/H3K9ac model indicated that the rs1323292 variant was predicted to be mainly located in promoter of blood cell types, especially in immune cell types. Table 2 provides the results from promoter analysis of six neurodegenerative disease variants using HaploReg (version 4.1).

**Table 2:** Promoter analysis of six neurodegenerative disease variants using HaploReg (version 4.1).

**SNP**	**15-state core model**	**25-state model**	**H3K4me1/H3K4me3 model**	**H3K27ac/H3K9ac model**
rs1323292	Dnd41 TCell Leukemia Cell Line		Primary T helper naive cells from peripheral blood, Primary T helper memory cells from peripheral blood 1, Primary T helper cells PMA-I stimulated, Primary T regulatory cells from peripheral blood, Colon Smooth Muscle, Duodenum Smooth Muscle, Dnd41 TCell Leukemia Cell Line	Primary mononuclear cells from peripheral blood, Liver, Duodenum Mucosa, Rectal Mucosa Donor 31, Dnd41 TCell Leukemia Cell Line
rs701006			Brain Cingulate Gyrus	Primary T helper naive cells from peripheral blood, Brain Angular Gyrus, Brain Cingulate Gyrus, Brain Inferior Temporal Lobe
rs1476679			Foreskin Fibroblast Primary Cells skin02	H1 Derived Mesenchymal Stem Cells, H9 Cells, iPS-15b Cells, Mesenchymal Stem Cell Derived Adipocyte Cultured Cells, Adipose Derived Mesenchymal Stem Cell Cultured Cells, Breast Myoepithelial Primary Cells, Mesenchymal Stem Cell Derived Chondrocyte Cultured Cells, Brain Anterior Caudate, Brain Inferior Temporal Lobe, Colonic Mucosa, Stomach Mucosa, NH-A Astrocytes Primary Cells
rs10838725			Brain Angular Gyrus, Colon Smooth Muscle, Small Intestine	Adipose Nuclei, Brain Anterior Caudate, Fetal Heart, Skeletal Muscle Male
rs76904798	NA	NA	NA	NA
rs3865444	hESC Derived CD184+ Endoderm Cultured Cells, Primary hematopoietic stem cells, HeLa-S3 Cervical Carcinoma Cell Line, K562 Leukemia Cells, Monocytes-CD14+ RO01746 Primary Cells	hESC Derived CD184+ Endoderm Cultured Cells, Primary monocytes from peripheral blood, Primary neutrophils from peripheral blood, Primary hematopoietic stem cells short term culture, Primary T helper memory cells from peripheral blood 2, Primary T helper naive cells from peripheral blood, Primary T helper naive cells from peripheral blood, Primary T helper cells from peripheral blood, Primary T CD8+ naive cells from peripheral blood, Primary hematopoietic stem cells G-CSF-mobilized Male, Adipose Nuclei, Liver, Fetal Brain Male, HeLa-S3 Cervical Carcinoma Cell Line	hESC Derived CD184+ Endoderm Cultured Cells, Primary neutrophils from peripheral blood, Primary hematopoietic stem cells, Primary hematopoietic stem cells short term culture, Primary hematopoietic stem cells G-CSF-mobilized Female, Primary hematopoietic stem cells G-CSF-mobilized Male, Primary mononuclear cells from peripheral blood, Liver, Brain Cingulate Gyrus, Brain Hippocampus Middle, Brain_Dorsolateral_Prefrontal_Cortex, Duodenum Mucosa, Fetal Adrenal Gland, Placenta, Fetal Thymus, Gastric, Lung, Pancreas, HeLa-S3 Cervical Carcinoma Cell Line, K562 Leukemia Cells, Monocytes-CD14+ RO01746 Primary Cells	Primary mononuclear cells from peripheral blood, HeLa-S3 Cervical Carcinoma Cell Line, K562 Leukemia Cells, Monocytes-CD14+ RO01746 Primary Cells

### DNAse Analysis

The DNAse analysis showed that rs1323292 variant was predicted to be mainly located in DNAse of blood cell types, especially in primary T cells from cord blood, primary T cells from peripheral blood, primary Natural Killer cells from peripheral blood, fetal Thymus, and small Intestine. The rs701006 variant was predicted to be mainly located in DNAse of Foreskin Melanocyte Primary Cells skin01 and Fetal Brain Female. The rs1476679 variant was predicted to be mainly located in DNAse of iPS DF 19.11 Cells, Breast variant Human Mammary Epithelial Cells (vHMEC), HSMM cell derived Skeletal Muscle Myotubes Cells. However, we did identify any rs10838725, rs76904798, rs3865444.

### eQTLs Analysis in CD14+ Monocytes

In the first dataset including up to 197 individuals, the results showed significant association of all these six genetic variants (rs3865444, rs1476679, rs10838725, rs76904798, rs1323292, and rs701006) with the expression of nearby genes including CD33, PILRB, NUP160, LRRK2, RGS1, and METTL21B. In addition, there are also some other genes. Here, we list the significant results with *P* < 0.01 and the corresponding false discovery rate (FDR) in Table 3.

**Table 3:** eQTL analysis of six neurodegenerative disease variants in CD14+ monocytes.

**SNP**	**Chr**	**Phenotype or probe ID**	**Gene**	***P*-value**	**FDR**	**Dataset**	**Reference**
rs1323292^*^	**1**	**ENSG00000090104.7**	**RGS1**	**3.07E-34**	**6.25E-31**	**CD14+ monocytes**	Chen et al., 2016
**rs701006**	**12**	**ENSG00000123427.11**	**METTL21B**	**3.67E-26**	**1.55E-24**	**CD14+ monocytes**	Chen et al., 2016
**rs1476679**	**7**	**ENSG00000121716.12**	**PILRB**	**6.74E-20**	**5.33E-19**	**CD14+ monocytes**	Chen et al., 2016
**rs10838725**	**11**	**ENSG00000030066.9**	**NUP160**	**5.73E-18**	**6.15E-18**	**CD14+ monocytes**	Chen et al., 2016
**rs76904798**	**12**	**ENSG00000188906.9**	**LRRK2**	**9.42E-15**	**3.48E-11**	**CD14+ monocytes**	Chen et al., 2016
**rs3865444**	**19**	**ENSG00000105383.10**	**CD33**	**5.26E-14**	**6.09E-11**	**CD14+ monocytes**	Chen et al., 2016
rs76904798	12	ENSG00000260943.1	RP11-476D10.1	3.84E-12	4.71E-11	CD14+ monocytes	Chen et al., 2016
rs3865444	19	ENSG00000268849.1	SIGLEC22P	1.72E-07	5.23E-05	CD14+ monocytes	Chen et al., 2016
rs10838725	11	ENSG00000134571.6	MYBPC3	1.79E-07	2.18E-07	CD14+ monocytes	Chen et al., 2016
rs10838725	11	ENSG00000110514.14	MADD	2.40E-06	5.68E-06	CD14+ monocytes	Chen et al., 2016
rs701006	12	ENSG00000123297.11	TSFM	2.48E-06	8.05E-05	CD14+ monocytes	Chen et al., 2016
rs1476679	7	ENSG00000221838.5	AP4M1	9.93E-06	5.90E-05	CD14+ monocytes	Chen et al., 2016
rs76904798	12	ENSG00000229899.1	AC084290.2	1.04E-05	7.97E-04	CD14+ monocytes	Chen et al., 2016
rs1476679	7	ENSG00000078319.7	PMS2P1	5.41E-05	8.76E-04	CD14+ monocytes	Chen et al., 2016
rs1476679	7	ENSG00000166997.3	CNPY4	8.00E-05	2.58E-03	CD14+ monocytes	Chen et al., 2016
rs701006	12	ENSG00000135506.11	OS9	2.50E-04	2.63E-02	CD14+ monocytes	Chen et al., 2016
rs1476679	7	ENSG00000087077.7	TRIP6	4.80E-04	7.17E-03	CD14+ monocytes	Chen et al., 2016
rs76904798	12	ENSG00000225342.1	AC079630.4	5.25E-04	2.49E-02	CD14+ monocytes	Chen et al., 2016
rs1476679	7	ENSG00000196411.5	EPHB4	8.54E-04	5.47E-03	CD14+ monocytes	Chen et al., 2016
rs701006	12	ENSG00000135439.6	AGAP2	8.61E-04	1.03E-02	CD14+ monocytes	Chen et al., 2016
rs3865444	19	ENSG00000142512.10	SIGLEC10	9.02E-04	1.47E-01	CD14+ monocytes	Chen et al., 2016
rs1476679	7	ENSG00000066923.12	STAG3	3.09E-03	1.10E-02	CD14+ monocytes	Chen et al., 2016
rs1476679	7	ENSG00000085514.10	PILRA	3.10E-03	7.72E-02	CD14+ monocytes	Chen et al., 2016
rs1476679	7	ENSG00000106290.10	TAF6	6.23E-03	3.42E-02	CD14+ monocytes	Chen et al., 2016
rs1476679	7	ENSG00000106330.7	MOSPD3	8.13E-03	3.15E-02	CD14+ monocytes	Chen et al., 2016
rs1476679	7	ENSG00000146830.8	GIGYF1	9.97E-03	4.92E-02	CD14+ monocytes	Chen et al., 2016
**rs701006**	**12**	**870056**	**METTL21B**	**4.79E-63**	**1.95E-59**	**CD14+ monocytes**	Fairfax et al., 2014
rs1476679	7	2190541	GATS	1.14E-20	5.73E-18	CD14+ monocytes	Fairfax et al., 2014
rs701006	12	5670577	TSPAN31	3.25E-15	1.04E-12	CD14+ monocytes	Fairfax et al., 2014
**rs11175655**	**12**	**3520291**	**LRRK2**	**6.66E-12**	**1.48E-09**	**CD14+ monocytes**	Fairfax et al., 2014
rs11175655	12	2490315	HS.306876	3.06E-10	5.51E-08	CD14+ monocytes	Fairfax et al., 2014
rs701006	12	5290239	XRCC6BP1	2.68E-09	4.22E-07	CD14+ monocytes	Fairfax et al., 2014
rs701006	12	3130102	TSFM	3.24E-09	5.04E-07	CD14+ monocytes	Fairfax et al., 2014
rs1476679	7	2810674	TRIM4	3.59E-09	5.54E-07	CD14+ monocytes	Fairfax et al., 2014
**rs1323298**	**1**	**4490176**	**RGS1**	**4.91E-09**	**7.45E-07**	**CD14+ monocytes**	Fairfax et al., 2014
**rs1476679**	**7**	**1570039**	**PILRB**	**9.85E-08**	**1.21E-05**	**CD14+ monocytes**	Fairfax et al., 2014
rs1476679	7	2030088	PILRA	1.88E-06	1.82E-04	CD14+ monocytes	Fairfax et al., 2014
**rs11175655**	**12**	**1820725**	**LRRK2**	**3.26E-05**	**2.40E-03**	**CD14+ monocytes**	Fairfax et al., 2014
rs1476679	7	2750168	AP4M1	4.30E-05	3.07E-03	CD14+ monocytes	Fairfax et al., 2014
rs1476679	7	5420672	PILRA	6.70E-03	1.83E-01	CD14+ monocytes	Fairfax et al., 2014
**rs701006**	**12**	**870056**	**METTL21B**	**6.35E-51**	**3.02E-47**	**CD14+ monocytes after 24-h LPS**	Fairfax et al., 2014
rs1476679	7	2190541	GATS	2.22E-26	2.39E-23	CD14+ monocytes after 24-h LPS	Fairfax et al., 2014
**rs11175655**	**12**	**3520291**	**LRRK2**	**2.70E-24**	**2.51E-21**	**CD14+ monocytes after 24-h LPS**	Fairfax et al., 2014
rs701006	12	2900725	CYP27B1	1.73E-19	1.12E-16	CD14+ monocytes after 24-h LPS	Fairfax et al., 2014
rs701006	12	650348	METTL1	2.68E-16	1.26E-13	CD14+ monocytes after 24-h LPS	Fairfax et al., 2014
rs11175655	12	2490315	HS.306876	7.94E-13	2.54E-10	CD14+ monocytes after 24-h LPS	Fairfax et al., 2014
rs1476679	7	2470577	ZCWPW1	2.31E-10	5.36E-08	CD14+ monocytes after 24-h LPS	Fairfax et al., 2014
**rs1323298**	**1**	**4490176**	**RGS1**	**3.98E-08**	**6.43E-06**	**CD14+ monocytes after 24-h LPS**	Fairfax et al., 2014
rs701006	12	5670577	TSPAN31	3.96E-07	5.30E-05	CD14+ monocytes after 24-h LPS	Fairfax et al., 2014
rs1476679	7	2810674	TRIM4	8.73E-07	1.09E-04	CD14+ monocytes after 24-h LPS	Fairfax et al., 2014
rs1476679	7	2030088	PILRA	1.29E-06	1.55E-04	CD14+ monocytes after 24-h LPS	Fairfax et al., 2014
rs701006	12	5290239	XRCC6BP1	1.75E-06	2.05E-04	CD14+ monocytes after 24-h LPS	Fairfax et al., 2014
rs701006	12	3130102	TSFM	4.19E-05	3.48E-03	CD14+ monocytes after 24-h LPS	Fairfax et al., 2014
rs701006	12	110180	ARHGAP9	7.18E-05	5.57E-03	CD14+ monocytes after 24-h LPS	Fairfax et al., 2014
rs701006	12	7380110	CDK4	3.91E-04	2.34E-02	CD14+ monocytes after 24-h LPS	Fairfax et al., 2014
rs1476679	7	4850487	UFSP1	6.45E-04	3.52E-02	CD14+ monocytes after 24-h LPS	Fairfax et al., 2014
rs1476679	7	5420672	PILRA	7.58E-04	4.01E-02	CD14+ monocytes after 24-h LPS	Fairfax et al., 2014
rs1476679	7	2750168	AP4M1	2.72E-03	1.06E-01	CD14+ monocytes after 24-h LPS	Fairfax et al., 2014
**rs701006**	**12**	**870056**	**METTL21B**	**1.11E-39**	**5.75E-36**	**CD14+ monocytes after 2-h LPS**	Fairfax et al., 2014
rs1476679	7	2030088	PILRA	1.41E-10	5.20E-08	CD14+ monocytes after 2-h LPS	Fairfax et al., 2014
rs1476679	7	5420672	PILRA	8.96E-10	2.90E-07	CD14+ monocytes after 2-h LPS	Fairfax et al., 2014
rs701006	12	2900725	CYP27B1	5.71E-09	1.61E-06	CD14+ monocytes after 2-h LPS	Fairfax et al., 2014
rs1476679	7	650040	PILRA	9.19E-09	2.49E-06	CD14+ monocytes after 2-h LPS	Fairfax et al., 2014
rs1476679	7	2190541	GATS	5.05E-08	1.18E-05	CD14+ monocytes after 2-h LPS	Fairfax et al., 2014
rs701006	12	650348	METTL1	1.16E-06	2.04E-04	CD14+ monocytes after 2-h LPS	Fairfax et al., 2014
**rs11175655**	**12**	**3520291**	**LRRK2**	**3.67E-06**	**5.69E-04**	**CD14+ monocytes after 2-h LPS**	Fairfax et al., 2014
rs701006	12	5670577	TSPAN31	7.85E-05	8.25E-03	CD14+ monocytes after 2-h LPS	Fairfax et al., 2014
rs701006	12	5290239	XRCC6BP1	1.69E-04	1.58E-02	CD14+ monocytes after 2-h LPS	Fairfax et al., 2014
rs1476679	7	1470195	MCM7	1.91E-04	1.75E-02	CD14+ monocytes after 2-h LPS	Fairfax et al., 2014
rs1476679	7	2810674	TRIM4	2.92E-04	2.48E-02	CD14+ monocytes after 2-h LPS	Fairfax et al., 2014
rs701006	12	7380110	CDK4	4.49E-03	1.88E-01	CD14+ monocytes after 2-h LPS	Fairfax et al., 2014
**rs701006**	**12**	**870056**	**METTL21B**	**1.30E-49**	**3.75E-46**	**CD14+ monocytes after exposure to IFN-γ**	Fairfax et al., 2014
**rs11175655**	**12**	**3520291**	**LRRK2**	**9.37E-23**	**5.54E-20**	**CD14+ monocytes after exposure to IFN-γ**	Fairfax et al., 2014
rs11175655	12	2490315	HS.306876	4.12E-18	1.69E-15	CD14+ monocytes after exposure to IFN-γ	Fairfax et al., 2014
rs701006	12	650348	METTL1	9.74E-13	2.33E-10	CD14+ monocytes after exposure to IFN-γ	Fairfax et al., 2014
**rs1323298**	**1**	**4490176**	**RGS1**	**1.72E-11**	**3.53E-09**	**CD14+ monocytes after exposure to IFN-γ**	Fairfax et al., 2014
rs701006	12	5670577	TSPAN31	3.56E-10	6.16E-08	CD14+ monocytes after exposure to IFN-γ	Fairfax et al., 2014
rs1476679	7	2030088	PILRA	8.04E-08	9.63E-06	CD14+ monocytes after exposure to IFN-γ	Fairfax et al., 2014
**rs11175655**	**12**	**1820725**	**LRRK2**	**2.34E-07**	**2.59E-05**	**CD14+ monocytes after exposure to IFN-γ**	Fairfax et al., 2014
rs701006	12	3130102	TSFM	8.53E-07	8.48E-05	CD14+ monocytes after exposure to IFN-γ	Fairfax et al., 2014
rs1476679	7	2810674	TRIM4	2.07E-06	1.90E-04	CD14+ monocytes after exposure to IFN-γ	Fairfax et al., 2014
rs1476679	7	2190541	GATS	2.44E-06	2.20E-04	CD14+ monocytes after exposure to IFN-γ	Fairfax et al., 2014
rs701006	12	5290239	XRCC6BP1	1.19E-05	9.19E-04	CD14+ monocytes after exposure to IFN-γ	Fairfax et al., 2014
rs701006	12	4610066	TMEM194A	6.51E-05	4.16E-03	CD14+ monocytes after exposure to IFN-γ	Fairfax et al., 2014
rs1476679	7	5420672	PILRA	1.29E-04	7.55E-03	CD14+ monocytes after exposure to IFN-γ	Fairfax et al., 2014
rs1476679	7	6200743	CNPY4	5.44E-04	2.55E-02	CD14+ monocytes after exposure to IFN-γ	Fairfax et al., 2014
rs1476679	7	3940750	LOC100134648	6.57E-04	2.98E-02	CD14+ monocytes after exposure to IFN-γ	Fairfax et al., 2014
rs701006	12	1030593	OS9	7.07E-04	3.17E-02	CD14+ monocytes after exposure to IFN-γ	Fairfax et al., 2014
rs701006	12	1580431	PIP4K2C	1.09E-03	4.49E-02	CD14+ monocytes after exposure to IFN-γ	Fairfax et al., 2014
rs1476679	7	2470577	ZCWPW1	1.23E-03	4.95E-02	CD14+ monocytes after exposure to IFN-γ	Fairfax et al., 2014
rs701006	12	2900725	CYP27B1	1.29E-03	5.13E-02	CD14+ monocytes after exposure to IFN-γ	Fairfax et al., 2014
rs701006	12	110180	ARHGAP9	4.40E-03	1.29E-01	CD14+ monocytes after exposure to IFN-γ	Fairfax et al., 2014
rs1476679	7	650040	PILRA	6.69E-03	1.72E-01	CD14+ monocytes after exposure to IFN-γ	Fairfax et al., 2014

* *The associations identified by Ryan and colleagues are bolded. The rs11175655 variant is in high LD with rs76904798 (r2 = 0.83, and D′ = 1), and rs1323298 is in high LD with rs1323292 (r2 = 0.92, and D′ = 0.97). Chr, chromosome; IFN-γ, interferon-γ; LPS, lipopolysaccharide*.

In the second dataset including 414 samples in the naive state, two genetic variants rs3865444 and rs10838725, as well as their proxy variants (*r*^2^ ≥ 0.8), are not available. The other four genetic variants or their proxy variants were significantly associated with the expression of nearby genes including PILRB, LRRK2, RGS1, and METTL21B. In brief, rs11175655 is in high LD with rs76904798 (*r*2 = 0.83, and D′ = 1), and rs1323298 is in high LD with rs1323292 (*r*2 = 0.92, and D′ = 0.97). Meanwhile, there also some other genes, as provided in Table 2. In addition to the naive state, some findings were also observed in other three states including 367 individuals after exposure to IFN-γ, 322 individuals after 24-h LPS, and 261 individuals after 2-h LPS, as provided in Table 3.

### QTLs Analysis in Human Peripheral Blood

In the two eQTL datasets in human peripheral blood, we again found significant association of all these six genetic variants (rs3865444, rs1476679, rs10838725, rs76904798, rs1323292, and rs701006) with the expression of nearby genes including CD33, PILRB, NUP160, LRRK2, RGS1, and METTL21B, as well as other genes (Table 4).

**Table 4:** eQTL analysis of six neurodegenerative disease variants in whole blood.

**SNP**	**Position (hg19)**	**Probe ID**	***P-*value**	**Sample size**	**Reference**
**rs1323292**	**chr1:192541021**	**RGS1**	**7.36E-05**	**2116**	Zhernakova et al., 2017
**rs701006**	**chr12:58106836**	**METTL21B**	**3.54E-224**	**2116**	Zhernakova et al., 2017
rs701006	chr12:58106836	TSFM;RP11-571M6.15	1.16E-40	2116	Zhernakova et al., 2017
rs701006	chr12:58106836	AVIL	1.80E-32	2116	Zhernakova et al., 2017
rs701006	chr12:58106836	TSFM	1.38E-25	2116	Zhernakova et al., 2017
rs701006	chr12:58106836	XRCC6BP1	3.26E-21	2116	Zhernakova et al., 2017
rs701006	chr12:58106836	OS9;RP11-571M6.7	4.95E-15	2116	Zhernakova et al., 2017
rs701006	chr12:58106836	MARCH9	2.78E-13	2116	Zhernakova et al., 2017
rs701006	chr12:58106836	AVIL;U6	4.08E-13	2116	Zhernakova et al., 2017
rs701006	chr12:58106836	TSPAN31	4.79E-13	2116	Zhernakova et al., 2017
rs701006	chr12:58106836	OS9	6.58E-12	2116	Zhernakova et al., 2017
rs701006	chr12:58106836	RP11-571M6.18	1.26E-10	2116	Zhernakova et al., 2017
rs701006	chr12:58106836	METTL1;METTL21B	4.04E-10	2116	Zhernakova et al., 2017
rs701006	chr12:58106836	CDK4;TSPAN31	1.90E-09	2116	Zhernakova et al., 2017
rs701006	chr12:58106836	METTL21B;RP11-571M6.15	2.35E-09	2116	Zhernakova et al., 2017
rs701006	chr12:58106836	METTL1;RP11-571M6.13	4.52E-05	2116	Zhernakova et al., 2017
rs701006	chr12:58106836	AGAP2	7.06E-05	2116	Zhernakova et al., 2017
rs701006	chr12:58106836	METTL1	7.42E-05	2116	Zhernakova et al., 2017
**rs1476679**	**chr7:100004446**	**PILRB**	**9.88E-127**	**2116**	Zhernakova et al., 2017
rs1476679	chr7:100004446	STAG3;PVRIG	3.96E-58	2116	Zhernakova et al., 2017
rs1476679	chr7:100004446	STAG3;GATS	4.26E-52	2116	Zhernakova et al., 2017
rs1476679	chr7:100004446	PILRA;PILRB	3.91E-37	2116	Zhernakova et al., 2017
rs1476679	chr7:100004446	STAG3;GATS;GATS	1.09E-32	2116	Zhernakova et al., 2017
rs1476679	chr7:100004446	STAG3;PVRIG;AC005071.1	2.05E-24	2116	Zhernakova et al., 2017
rs1476679	chr7:100004446	PILRB;CTB-161A2.4	4.97E-22	2116	Zhernakova et al., 2017
rs1476679	chr7:100004446	GAL3ST4	1.21E-18	2116	Zhernakova et al., 2017
rs1476679	chr7:100004446	ZCWPW1	1.38E-11	2116	Zhernakova et al., 2017
rs1476679	chr7:100004446	MOSPD3	2.82E-09	2116	Zhernakova et al., 2017
rs1476679	chr7:100004446	GPC2	2.92E-08	2116	Zhernakova et al., 2017
rs1476679	chr7:100004446	PILRB;CTB-161A2.3	5.97E-08	2116	Zhernakova et al., 2017
rs1476679	chr7:100004446	TFR2	1.98E-06	2116	Zhernakova et al., 2017
rs1476679	chr7:100004446	PILRA	6.63E-06	2116	Zhernakova et al., 2017
rs1476679	chr7:100004446	C7orf61	4.12E-05	2116	Zhernakova et al., 2017
rs1476679	chr7:100004446	TSC22D4;C7orf61	4.34E-05	2116	Zhernakova et al., 2017
rs1476679	chr7:100004446	PPP1R35;RP11-758P17.2	5.99E-05	2116	Zhernakova et al., 2017
rs10838725	chr11:47557871	MYBPC3	3.05E-47	2116	Zhernakova et al., 2017
rs10838725	chr11:47557871	C1QTNF4	6.18E-44	2116	Zhernakova et al., 2017
rs10838725	chr11:47557871	MADD	8.32E-25	2116	Zhernakova et al., 2017
rs10838725	chr11:47557871	FNBP4	2.20E-18	2116	Zhernakova et al., 2017
rs10838725	chr11:47557871	FNBP4;Y_RNA	2.92E-15	2116	Zhernakova et al., 2017
rs10838725	chr11:47557871	RP11-750H9.5	5.00E-13	2116	Zhernakova et al., 2017
rs10838725	chr11:47557871	SLC39A13	1.16E-09	2116	Zhernakova et al., 2017
rs10838725	chr11:47557871	RAPSN	1.33E-07	2116	Zhernakova et al., 2017
**rs76904798**	**chr12:40614434**	**LRRK2**	**1.47E-52**	**2116**	Zhernakova et al., 2017
rs76904798	chr12:40614434	RP11-476D10.1	1.93E-45	2116	Zhernakova et al., 2017
**rs3865444**	**chr19:51727962**	**CD33**	**1.58E-44**	**2116**	Zhernakova et al., 2017
rs3865444	chr19:51727962	SIGLEC22P	5.55E-24	2116	Zhernakova et al., 2017
rs3865444	chr19:51727962	VSIG10L	5.74E-06	2116	Zhernakova et al., 2017
rs1323292	chr1:192541021	SLC41A1	3.44E-05	5257	Joehanes et al., 2017
rs1323292	chr1:192541021	P4HTM	5.15E-05	5257	Joehanes et al., 2017
rs701006	chr12:58106836	AVIL;PP12719; LOC100653271	2.45E-38	5257	Joehanes et al., 2017
rs701006	chr12:58106836	METTL21B	9.31E-31	5257	Joehanes et al., 2017
rs701006	chr12:58106836	ARHGAP9	2.71E-09	5257	Joehanes et al., 2017
rs701006	chr12:58106836	TSPAN31	7.01E-07	5257	Joehanes et al., 2017
rs701006	chr12:58106836	OS9	7.77E-07	5257	Joehanes et al., 2017
rs701006	chr12:58106836	XRCC6BP1	2.87E-06	5257	Joehanes et al., 2017
rs701006	chr12:58106836	GLI1	3.96E-06	5257	Joehanes et al., 2017
rs701006	chr12:58106836	TSFM	7.43E-05	5257	Joehanes et al., 2017
rs1476679	chr7:100004446	PILRB	6.16E-69	5257	Joehanes et al., 2017
rs1476679	chr7:100004446	PVRIG;STAG3	1.23E-44	5257	Joehanes et al., 2017
rs1476679	chr7:100004446	GATS	1.56E-26	5257	Joehanes et al., 2017
rs1476679	chr7:100004446	EPHB4	4.30E-12	5257	Joehanes et al., 2017
rs1476679	chr7:100004446	TRIM4	1.87E-07	5257	Joehanes et al., 2017
rs1476679	chr7:100004446	PILRA	1.82E-06	5257	Joehanes et al., 2017
rs1476679	chr7:100004446	CNPY4	1.00E-05	5257	Joehanes et al., 2017
rs1476679	chr7:100004446	MAN2B1	1.38E-05	5257	Joehanes et al., 2017
rs1476679	chr7:100004446	ZKSCAN1	1.84E-05	5257	Joehanes et al., 2017
rs1476679	chr7:100004446	OR2AE1	2.45E-05	5257	Joehanes et al., 2017
rs1476679	chr7:100004446	C3orf30;IGSF11	4.87E-05	5257	Joehanes et al., 2017
rs1476679	chr7:100004446	AQP4	5.83E-05	5257	Joehanes et al., 2017
rs10838725	chr11:47557871	MADD	7.68E-32	5257	Joehanes et al., 2017
rs10838725	chr11:47557871	PTPRJ	1.06E-22	5257	Joehanes et al., 2017
rs10838725	chr11:47557871	MTCH2	1.64E-12	5257	Joehanes et al., 2017
rs10838725	chr11:47557871	MYBPC3	6.88E-11	5257	Joehanes et al., 2017
rs10838725	chr11:47557871	SLC39A13	5.96E-06	5257	Joehanes et al., 2017
rs10838725	chr11:47557871	SBF2	1.90E-05	5257	Joehanes et al., 2017
rs10838725	chr11:47557871	NDUFS3	2.86E-05	5257	Joehanes et al., 2017
rs10838725	chr11:47557871	DKK4	3.64E-05	5257	Joehanes et al., 2017
**rs10838725**	**chr11:47557871**	**NUP160**	**6.14E-05**	**5257**	Joehanes et al., 2017
rs10838725	chr11:47557871	FCER2	7.50E-05	5257	Joehanes et al., 2017
rs76904798	chr12:40614434	TANK	3.77E-05	5257	Joehanes et al., 2017
rs3865444	chr19:51727962	ETFB	2.21E-07	5257	Joehanes et al., 2017
rs3865444	chr19:51727962	PITPNM2	5.24E-05	5257	Joehanes et al., 2017

*The associations identified by Ryan and colleagues are bolded*.

## Discussion

In recent years, large-scale GWAS datasets have identified 94 genes associated with Alzheimer's disease, Parkinson's disease, or multiple sclerosis (Ryan et al., 2017). However, it is still unclear how these variants functionally affect the underlying neurodegenerative disease pathogenesis. Growing evidence shows that genetic variants may affect disease risk by regulating gene expression (Bao et al., 2015; Liu et al., 2015, 2016, 2017a; Hu et al., 2017). Ryan and colleagues applied a human MDMi cellular model, and conducted an eQTL analysis to evaluate the effects of these neurodegenerative disease variants (Ryan et al., 2017).

In summary, Ryan et al. identified that six neurodegenerative disease variants were associated with disease susceptibility, and could alter the expression of six nearby genes. Both rs1476679 and rs76904798 variants could only regulate the expression of PILRB and LRRK2 in the MDMi cells, but not in human peripheral blood monocytes (Ryan et al., 2017). Ryan et al. concluded that the differentiation of monocytes into microglia-like cells could cause the acquisition of a cellular state, which could reveal the functional consequences of certain genetic variants (Ryan et al., 2017). Ryan et al. provided an *in vitro* translational tool to generate microglia-like cells quickly and easily from adult blood, and could be useful for exploring microglia function and dysfunction (Ryan et al., 2017). However, Ryan et al. just selected single eQTL dataset in human peripheral blood monocytes without any replication.

Here, we first performed an enhancer analysis of these six neurodegenerative disease variants. The results showed that these six genetic variants were predicted to be mainly located in enhancers of blood cell types, especially in CD14+ monocytes. The promoter analysis showed that rs3865444 and rs1323292 variants were predicted to be mainly located in promoter of blood cell types, especially in immune cell types. Hence, we further evaluated these six genetic variants using multiple eQTL datasets in human peripheral blood immune cell CD14+ monocytes. The results that showed that rs1476679 and rs76904798 variants or their proxy variants could significantly regulate the expression of PILRB and LRRK2 in immune cell CD14+ monocytes and human peripheral blood.

In summary, we believe that these findings provide important supplementary information about the regulatory mechanisms by which both variants influence PILRB and LRRK2 gene expression and neurodegenerative disease risk.

## Author Contributions

BS and HY conceived and initiated the project. JS and YH analyzed the data, and wrote the first draft of the manuscript. YZ, LW, and LL contributed to the interpretation of the results and critical revision of the manuscript for important intellectual content and approved the final version of the manuscript.

### Conflict of Interest Statement

The authors declare that the research was conducted in the absence of any commercial or financial relationships that could be construed as a potential conflict of interest.
